# Length of stay and odds of MRSA acquisition: a dose–response relationship?

**DOI:** 10.1017/S0950268819001110

**Published:** 2019-06-21

**Authors:** H. Y. Loke, W. M. Kyaw, M. I. C. Chen, J. W. Lim, B. Ang, A. Chow

**Affiliations:** 1 Department of Clinical Epidemiology, Office of Clinical Epidemiology, Analytics, and Knowledge, Tan Tock Seng Hospital, Singapore; 2 Saw Swee Hock School of Public Health, National University of Singapore, Singapore; 3 Department of Infectious Diseases, Tan Tock Seng Hospital, Singapore

**Keywords:** Bacterial infections, hospital-acquired (nosocomial) infections, methicillin-resistant *S. aureus* (MRSA)

## Abstract

The association between methicillin-resistant *Staphylococcus aureus* (MRSA) colonisation and/or infection with increased morbidity and mortality among hospital patients has long been recognised. We sought to build on previous studies to identify modifiable risk factors associated with the acquisition of MRSA colonisation and infection by conducting a retrospective cohort study on patients admitted through the Emergency Department of an acute tertiary-care general hospital in Singapore which implemented universal on-admission MRSA screening. Patients were assigned to the acquisition or non-acquisition group depending on whether they acquired MRSA during their admission. We used logistic regression models with a patient being in the acquisition group as the binary outcome to identify factors associated with MRSA acquisition. A total of 1302 acquisition and 37 949 non-acquisition group patients were analysed. Fifteen variables were included in the multivariate model. A dose–response relationship between length of stay and odds of MRSA acquisition was observed, with a length of stay 3 weeks or more (Adj OR 11.78–57.36, all *P* < 0.001) being the single biggest predictor of MRSA acquisition. Other variables significantly associated with MRSA acquisition were: male gender, age 65 or greater, previous MRSA colonisation or infection, exposure to certain antibiotics and surgery, and history of diabetes.

## Introduction

Methicillin-resistant *Staphylococcus aureus* (MRSA) is a bacterium resistant to many commonly used antibiotics, and a leading cause of nosocomial infection. MRSA may be acquired through contact with a contaminated source, and can lead to asymptomatic colonisation. Compared to methicillin-susceptible *S. aureus*, colonisation with MRSA is more likely to be associated with infection, morbidity and mortality [1,2].

Hence, the identification of MRSA acquisition in the acute tertiary hospital setting is crucial, as many environmental and patient factors associated with MRSA acquisition are commonly present in such a care context. Previously identified risk factors for MRSA acquisition include exposure to patients colonised or infected with MRSA and exposure to certain care settings such as the intensive care unit [3]. Previously reported patient risk factors include advanced age, male gender, immunocompromised state, exposure to surgery, certain classes of antibiotics and longer length of stay [4,5]. Many of these studies have included small study populations and have used study designs that would not allow for causal inference.

To better elucidate causal factors for MRSA acquisition in the acute tertiary hospital setting, a large longitudinal patient cohort design is preferred. The objective of this study was to identify modifiable factors associated with MRSA acquisition in a large acute tertiary hospital, using a patient cohort universally screened for MRSA on-admission and followed up longitudinally for clinical MRSA infection or MRSA screening at discharge.

## Methods

### Study type and study population

We assembled a cohort of patients admitted to Tan Tock Seng Hospital (TTSH) in Singapore, an acute tertiary-care general hospital with 27 clinical specialties and 1600 beds. The study cohort consisted of patients admitted to TTSH through the hospital's Emergency Department between 16 January 2012 and 1 January 2014, 16 years or older, and had MRSA screening test done on admission and either: developed clinical MRSA infection during admission, or had MRSA screening test done at discharge. The hospital has a universal on-entry MRSA screening programme: an MRSA screening is performed on all agreeing patients admitted from the Emergency Department unless a contraindication, such as facial injury, is present. Patients screened positive for MRSA are admitted into designated MRSA wards, and patients screened negative into non-MRSA wards.

Patients were defined as having acquired MRSA (‘acquisition group’) if they had a negative MRSA screening test result on admission and either a positive MRSA screening test result at discharge or MRSA cultured in a clinical sample taken more than 48 h post-admission. Patients were defined as not having acquired MRSA (‘non-acquisition group’) if they had negative MRSA screening test results both on admission and at discharge. If a patient had multiple admissions during the study period, only the first admission within the study period was included in the analysis. Patients were excluded from the study if they (a) developed MRSA infection within 48 h from the time of admission, or (b) did not have a discharge MRSA screening result and did not develop MRSA infection during the admission.

A sensitivity analysis was performed on patients who acquired MRSA colonisation only, by removing all patients who developed MRSA infection more than 48 h after admission from the acquisition group.

On admission, nasal swabs for MRSA screening were taken at the Emergency Department prior to ward transfer. At discharge, pooled swabs from the nares, axillae and groin for MRSA screening were taken within 24 h prior to the patients' discharge from hospital. MRSA screening on-admission was performed using the GeneXpert real-time PCR test [6], and screening at-discharge was done via culture using selective chromogenic agar plates (Brilliance MRSA 2 agar, Oxoid, UK). The sensitivity and specificity of the GeneXpert real-time PCR test were comparable to the conventional culture, as previously reported [7,8].

### Data sources and data collection

Information on demographics, comorbidities, hospitalisation history, the current admission, antibiotics and surgical exposures was retrieved from TTSH's operations databases and electronic medical records. Microbiological data were extracted from TTSH's laboratory database.

We sought to categorise surgical procedures by the degree of anatomical disruption. We used the Table of Surgical Procedures (TOSP) published by Singapore's Ministry of Health as a proxy for this purpose to assign procedures to minor, moderate and major categories. The TOSP covers about 1600 procedures classified into 21 tables based on the complexity of the surgery. The TOSP assigns a table ranking to surgical procedures from 1A (least complex) to 7C (most complex). We assigned procedures from Tables 1A to 3C, 4A to 5C and 6A to 7C to the minor, moderate and major surgery categories, respectively.

### Statistical analysis

Data were extracted, coded, then analysed using Stata 13 (Stata Corp., College Station, TX, USA). Crude odds ratios and 95% confidence intervals for all variables were obtained by using logistic regression.

Variables were tested for multicollinearity. Variables with collinearity were not simultaneously considered in the final model. We used logistic regression with a patient being in the acquisition group as a binary outcome to identify factors associated with MRSA acquisition. We included confounding variables, as well as factors which were found in other studies to be associated with MRSA acquisition, in the statistical model, such as gender, age, previous history of MRSA colonisation or infection in the past year, length of stay, major medical conditions, exposure to antibiotics by class and exposure to surgery.

We also included in the model potential confounding variables specific to the study environment, such as specialty discharged from, as well as study quartile the patient was admitted in; as the beginning of the study period (16 January 2012) coincided with the introduction of universal MRSA screening for admissions from the Emergency Department, we divided the study period into quartiles to examine whether a patient's admission quartile during the study period was associated with being in the MRSA acquisition group.

### Study approval

This study was approved by the domain-specific review board (DSRB) of the National Healthcare Group, Singapore (NHG DSRB Ref 2012/01119).

## Results

A total of 39 251 patients were included in the study, of which 1302 (3.3%) were classified into the acquisition group.

Table 1 presents the baseline characteristics of the acquisition and non-acquisition groups. Patients in the acquisition group were more likely to be male (OR 1.34, 95% CI 1.19–1.50), 65 years and above (OR 3.94, 95% CI 3.44–4.51), and have a history of MRSA colonisation or infection in the preceding admission over the last year (OR 7.59, 95% CI 6.16–9.35). Patients in the acquisition group were also more likely to have a history of cancer (OR 1.84, 95% CI 1.51–2.24), cerebrovascular disease (OR 1.79, 95% CI 1.44–2.22), diabetes (OR 1.85, 95% CI 1.57–2.19) and renal impairment (OR 2.10, 95% CI 1.65–2.66).

**Table 1.** Baseline characteristics and univariate comparisons of the acquisition and non-acquisition groups (*n* = 39 251)

Characteristic	Acquisition group *n* = 1302		Non-acquisition group *n* = 37 949		*P*	OR (95%CI)
	*n*	%	*n*	%		
Male	760	58.4	19 429	51.2	<0.001	1.34 (1.19–1.50)
Age ⩾65	1032	79.3	18 698	49.3	<0.001	3.94 (3.44–4.51)
History of MRSA colonisation or infection within 1 year of admission	118	9.1	492	1.3	<0.001	7.59 (6.16–9.35)
Cancer^a^	109	8.4	1901	5.0	<0.001	1.84 (1.51–2.24)
Cardiac disease^b^	80	6.1	1994	5.3	0.159	1.18 (0.94–1.49)
Cerebrovascular disease^c^	94	7.2	1583	4.2	<0.001	1.79 (1.44–2.22)
Diabetes	167	12.8	2789	7.3	<0.001	1.85 (1.57–2.19)
Pulmonary disease^d^	57	4.4	1856	4.9	0.398	0.89 (0.68–1.17)
Renal impairment	77	5.9	1105	2.9	<0.001	2.10 (1.65–2.66)
Liver disease^e^	16	1.2	425	1.1	0.714	1.10 (0.66–1.82)

a Does not include skin cancers.

b Includes history of myocardial infarction and congestive cardiac failure.

c Includes transient ischaemic attack.

d Includes asthma, chronic obstructive pulmonary disease, emphysema, bronchiectasis.

e Includes liver failure, fibrosis, portal hypertension. Does not include viral hepatitis.

Table 2 presents results from the univariate analysis of epidemiologic and clinical factors among patients in the acquisition and non-acquisition groups. Patients in the acquisition group were more likely to have been exposed to antibiotics during the current admission. This was significant for every antibiotic class except monobactam. Additionally, patients in the acquisition group were more likely to have been exposed to all subcategories of surgery. Patients discharged from dermatology, geriatric medicine, palliative medicine, rehabilitation medicine and plastic surgery were more likely to have acquired MRSA than patients discharged from general medicine. Being admitted in the third and fourth study quartiles was significantly associated with reducing odds of having acquired MRSA during the admission on univariate analysis. Expectedly, an increasing association between a longer length of stay and MRSA acquisition was observed.

**Table 2.** Univariate analysis of factors among patients in the acquisition and non-acquisition groups (*n* = 39 251)

	Acquisition group *n* = 1302		Non-acquisition group *n* = 37 949			
	*n*	%	*N*	%	*P*	OR (95%CI)
Antibiotic exposure by antibiotic class						
Aminoglycoside	141	10.8	720	1.9	<0.001	6.28 (5.19–7.59)
BS cephalosporin^a^	242	18.6	3703	9.8	<0.001	2.11 (1.83–2.44)
NS cephalosporin^b^	34	2.6	305	0.8	<0.001	3.31 (2.31–4.74)
BS penicillin^a^	283	21.7	1728	4.6	<0.001	5.82 (5.06–6.70)
NS penicillin^b^	502	38.6	4889	12.9	<0.001	4.24 (3.78–4.76)
Carbapenem	144	11.1	545	1.4	<0.001	8.53 (7.04–10.35)
Fluoroquinolone	206	15.8	1457	3.8	<0.001	4.71 (4.02–5.51)
Monobactam	1	0.1	11	0.0	0.351	2.65 (0.34–20.55)
Vancomycin	242	18.6	1174	3.1	<0.001	7.15 (6.15–8.32)
Other	256	19.7	3900	10.3	<0.001	2.14 (1.86–2.46)
Surgery exposure by urgency and complexity^c^						
Elective minor	101	7.8	1832	4.8	<0.001	1.66 (1.35–2.04)
Elective moderate	40	3.1	605	1.6	<0.001	1.95 (1.41–2.71)
Elective major	14	1.1	134	0.4	<0.001	3.07 (1.76–5.33)
Emergency minor	203	15.6	4961	13.1	0.008	1.23 (1.05–1.43)
Emergency moderate	71	5.5	1414	3.7	0.001	1.49 (1.17–1.90)
Emergency major	21	1.6	110	0.3	<0.001	5.64 (3.52–9.02)
Discharge specialty						
Cardiology	15	1.2	2577	6.8	<0.001	0.15 (0.95–0.27)
Dermatology	10	0.8	86	0.2	0.001	3.18 (1.63–6.17)
Others^d^	13	1.0	977	2.6	<0.001	0.36 (0.21–0.64)
Short stay	8	0.6	2021	5.3	<0.001	0.11 (0.05–0.22)
Gastroenterology	26	2.0	1229	3.2	0.008	0.58 (0.39–0.87)
Internal medicine	313	24.0	8551	22.5	**Ref**	**Ref**
Geriatric medicine	381	29.3	1978	5.2	<0.001	5.26 (4.50–6.16)
General surgery	121	9.3	4896	12.9	<0.001	0.68 (0.55–0.84)
Heart failure services	13	1.0	540	1.4	0.144	0.66 (0.38–1.15)
Infectious disease	17	1.3	1729	4.6	<0.001	0.27 (0.16–0.44)
Orthopaedic surgery	95	7.3	3254	8.6	0.057	0.80 (0.63–1.01)
Palliative care	10	0.8	65	0.2	<0.001	4.20 (2.14–8.26)
Psychiatry	5	0.4	112	0.3	0.667	1.22 (0.49–3.01)
Rheumatology	12	0.9	577	1.5	0.057	0.57 (0.32–1.02)
Rehabilitation medicine	80	6.1	246	0.6	<0.001	8.89 (6.74–11.71)
Nephrology	16	1.2	361	1.0	0.465	1.21 (0.72–2.02)
Respiratory medicine	37	2.8	2217	5.8	<0.001	0.46 (0.32–0.64)
Neurology	66	5.1	4540	12.0	<0.001	0.40 (0.30–0.52)
Neurosurgery	33	2.5	1125	3.0	0.233	0.80 (0.56–1.15)
Plastic surgery	5	0.4	30	0.1	0.002	4.55 (1.75–11.81)
Urology	26	2.0	838	2.2	0.425	0.85 (0.56–1.27)
Admission quartile						
Q1	412	31.6%	10 509	27.7	**Ref**	**Ref**
Q2	359	27.6%	9522	25.1	0.595	0.96 (0.83–1.11)
Q3	294	22.6%	9101	24.0	0.013	0.82 (0.71–0.96)
Q4	237	18.2%	8817	23.2	<0.001	0.69 (0.58–0.81)
Length of stay (days)						
1–6	260	20.0%	27 638	72.8	**Ref**	**Ref**
7–13	275	21.1%	7047	18.6	<0.001	4.15 (3.49–4.92)
14–20	181	13.9%	1710	4.5	<0.001	11.25 (9.25–13.69)
21–27	146	11.2%	689	1.8	<0.001	22.53 (18.14–27.97)
28–34	110	8.4%	321	0.8	<0.001	36.43 (28.41–46.71)
35–41	66	5.1%	188	0.5	<0.001	37.32 (27.48–50.68)
42–48	53	4.1%	119	0.3	<0.001	47.34 (33.50–66.91)
49–55	39	3.0%	63	0.2	<0.001	65.80 (43.34–99.91)
56–62	36	2.8%	41	0.1	<0.001	93.34 (58.69–148.45)
63 +	136	10.4%	133	0.4	<0.001	108.70 (83.11–142.16)

a BS, broad spectrum.

b NS, narrow spectrum.

c Minor/moderate/major classification based on Table of Surgical Procedures: tables 1–3 = minor, tables 4 and 5 = moderate, tables 6 and 7 = major.

d Specialty departments with <5 patients in the acquisition cohort were aggregated under Others. These specialties included haematology, otorhinolaryngology, ophthalmology, hand surgery, medical oncology and the tuberculosis unit.

On multivariate analysis (Table 3), male gender, age 65 years or greater and history of MRSA colonisation or infection in the previous year were independently associated with MRSA acquisition. The following antibiotics were positively associated with MRSA acquisition: aminoglycosides, narrow-spectrum penicillins, fluoroquinolones and vancomycin (*P* < 0.001).

**Table 3.** Multivariable analysis of factors associated with MRSA acquisition (n = 39 251)

	*P*	OR, 95%CI	*P*	OR (95%CI)
	Univariate		Multivariate	
Subject characteristic				
Male	<0.001	1.34 (1.19–1.50)	<0.001	1.62 (1.43–1.84)
Age ⩾65	<0.001	3.94 (3.44–4.51)	<0.001	1.84 (1.57–2.16)
History of MRSA colonisation or infection within 1 year of admission	<0.001	7.59 (6.16–9.35)	<0.001	6.39 (4.98–8.22)
Cancer	<0.001	1.84 (1.51–2.24)	0.678	1.05 (0.82–1.34)
Cardiac disease	0.159	1.18 (0.94–1.49)	0.402	0.89 (0.67–1.17)
Cerebrovascular disease	<0.001	1.79 (1.44–2.22)	0.831	1.03 (0.80–1.33)
Diabetes	<0.001	1.85 (1.57–2.19)	0.004	1.34 (1.10–1.64)
Pulmonary disease	0.398	0.89 (0.68–1.17)	0.385	1.15 (0.84–1.59)
Renal impairment	<0.001	2.10 (1.65–2.66)	0.895	1.02 (0.76–1.37)
Liver disease	0.714	1.10 (0.66–1.82)	0.672	0.89 (0.51–1.55)
Antibiotic exposure				
Aminoglycoside	<0.001	6.28 (5.19–7.59)	0.020	1.34 (1.05–1.71)
BS cephalosporin	<0.001	2.11 (1.83–2.44)	0.922	1.01 (0.84–1.21)
NS cephalosporin	<0.001	3.31 (2.31–4.74)	0.378	0.82 (0.53–1.27)
BS penicillin	<0.001	5.82 (5.06–6.70)	0.226	0.88 (0.72–1.08)
NS penicillin	<0.001	4.24 (3.78–4.76)	<0.001	1.36 (1.17–1.58)
Carbapenem	<0.001	8.53 (7.04–10.35)	0.173	1.19 (0.93–1.53)
Fluoroquinolone	<0.001	4.71 (4.02–5.51)	<0.001	1.78 (1.46–2.16)
Monobactam	0.351	2.65 (0.34–20.55)	0.397	0.38 (0.04–3.56)
Vancomycin	<0.001	7.15 (6.15–8.32)	0.002	1.45 (1.15–1.83)
Other	<0.001	2.14 (1.86–2.46)	0.646	0.96 (0.81–1.14)
Surgery exposure				
Elective minor	<0.001	1.66 (1.35–2.04)	0.103	0.81 (0.62–1.04)
Elective moderate	<0.001	1.95 (1.41–2.71)	0.418	0.85 (0.57–1.26)
Elective major	<0.001	3.07 (1.76–5.33)	0.059	0.51 (0.25–1.03)
Emergency minor	0.008	1.23 (1.05–1.43)	0.970	1.00 (0.83–1.22)
Emergency moderate	0.001	1.49 (1.17–1.90)	0.139	0.80 (0.59–1.08)
Emergency major	<0.001	5.64 (3.52–9.02)	0.052	1.83 (1.00–3.38)
Discharge department				
Cardiology	<0.001	0.15 (0.95–0.27)	<0.001	0.36 (0.21–0.61)
Dermatology	0.001	3.18 (1.63–6.17)	<0.001	3.83 (1.84–7.98)
Others	<0.001	0.36 (0.21–0.64)	<0.001	0.28 (0.15–0.51)
Short stay	<0.001	0.11 (0.05–0.22)	0.006	0.36 (0.18–0.75)
Gastroenterology	0.008	0.58 (0.39–0.87)	0.484	0.85 (0.54–1.34)
Internal medicine	**Ref**	**Ref**	**Ref**	**Ref**
Geriatric medicine	<0.001	5.26 (4.50–6.16)	<0.001	1.57 (1.30–1.90)
General surgery	<0.001	0.68 (0.55–0.84)	0.240	0.86 (0.67–1.11)
Heart failure services	0.144	0.66 (0.38–1.15)	0.857	1.06 (0.57–1.96)
Infectious disease	<0.001	0.27 (0.16–0.44)	0.002	0.42 (0.24–0.72)
Orthopaedic surgery	0.057	0.80 (0.63–1.01)	0.003	0.67 (0.51–0.87)
Palliative care	<0.001	4.20 (2.14–8.26)	0.957	1.02 (0.47–2.20)
Psychiatry	0.667	1.22 (0.49–3.01)	0.880	0.93 (0.35–2.46)
Rheumatology	0.057	0.57 (0.32–1.02)	0.613	1.17 (0.64–2.15)
Rehabilitation medicine	<0.001	8.89 (6.74–11.71)	0.119	1.31 (0.93–1.85)
Nephrology	0.465	1.21 (0.72–2.02)	0.411	0.78 (0.43–1.42)
Respiratory medicine	<0.001	0.46 (0.32–0.64)	<0.001	0.40 (0.27–0.59)
Neurology	<0.001	0.40 (0.30–0.52)	<0.001	0.59 (0.44–0.79)
Neurosurgery	0.233	0.80 (0.56–1.15)	0.103	0.71 (0.46–1.07)
Plastic surgery	0.002	4.55 (1.75–11.81)	0.037	4.17 (1.09–15.91)
Urology	0.425	0.85 (0.56–1.27)	0.616	1.12 (0.72–1.75)
Admission quartile				
Q1	**Ref**	**Ref**	**Ref**	**Ref**
Q2	0.595	0.96 (0.83–1.11)	0.921	0.99 (0.84–1.17)
Q3	0.013	0.82 (0.71–0.96)	0.239	0.90 (0.76–1.07)
Q4	<0.001	0.69 (0.58–0.81)	0.020	0.79 (0.66–0.96)
Length of stay (days)				
1–6	**Ref**	**Ref**	**Ref**	**Ref**
7–13	<0.001	4.15 (3.49–4.92)	<0.001	2.69 (2.23–3.25)
14–20	<0.001	11.25 (9.25–13.69)	<0.001	6.70 (5.35–8.38)
21–27	<0.001	22.53 (18.14–27.97)	<0.001	11.78 (9.13–15.21)
28–34	<0.001	36.43 (28.41–46.71)	<0.001	19.02 (14.17–25.53)
35–41	<0.001	37.32 (27.48–50.68)	<0.001	18.88 (13.21–26.97)
42–48	<0.001	47.34 (33.50–66.91)	<0.001	25.40 (17.09–37.76)
49–55	<0.001	65.80 (43.34–99.91)	<0.001	31.45 (19.71–50.18)
56–62	<0.001	93.34 (58.69–148.45)	<0.001	46.93 (27.60–79.80)
63+	<0.001	108.70 (83.11–142.16)	<0.001	57.36 (40.75–80.73)

A significant dose–response was observed with the length of hospital stay, with the odds for MRSA acquisition among patients hospitalised for 7–13 days being 2.7 times that of those with 1–6 days' stay (Adj OR 2.69, 95% CI 2.23–3.25) increasing to more than 50 times in patients hospitalised for more than 63 days (*P* < 0.001). Patients being discharged from dermatology (Adj OR 3.83, 95% CI 1.84–7.98), geriatric medicine (Adj OR 1.57, 95% CI 1.30–1.90) and plastic surgery (Adj OR 4.17, 95% CI 1.09–15.91) departments remain at increased odds of MRSA acquisition, even after accounting for age, gender, comorbidities, surgical and antibiotic exposures, admission quartile and length of stay. Having a major emergency surgery was marginally significantly associated with MRSA acquisition (Adj OR 1.83, 95% CI 1.00–3.38).

We performed a sensitivity analysis by repeating the multivariate analysis after excluding all patients in the acquisition group who had a positive MRSA culture more than 48 h from admission (Table 4). In the sensitivity analysis, vancomycin exposure was no longer significantly associated with MRSA acquisition (Adj OR 1.10, 95% CI 0.85–1.42), and the previously observed significant dose–response relationship was still present, albeit to a smaller extent. This suggests that the earlier observed association may have been due to vancomycin administered to patients for treatment of MRSA infection, as changes in the effect sizes of the remaining antibiotic classes were minimal.

**Table 4.** Sensitivity analysis of antibiotic exposure and length of stay between patients in the non-acquisition and acquisition (of MRSA colonisation only) groups

	*P*	OR (95%CI)	*P*	OR (95%CI)
	Multivariate (*n* = 39 251)		Multivariate (sensitivity analysis; *n* = 39 113)	
Antibiotic exposure				
Aminoglycoside	0.020	1.34 (1.05–1.71)	0.043	1.31 (1.01–1.71)
BS cephalosporin^a^	0.922	1.01 (0.84–1.21)	0.752	1.03 (0.85–1.25)
NS cephalosporin^b^	0.378	0.82 (0.53–1.27)	0.239	0.76 (0.47–1.20)
BS penicillin^a^	0.226	0.88 (0.72–1.08)	0.322	0.90 (0.72–1.11)
NS penicillin^b^	<0.001	1.36 (1.17–1.58)	<0.001	1.39 (1.18–1.62)
Carbapenem	0.173	1.19 (0.93–1.53)	0.428	1.12 (0.85–1.47)
Fluoroquinolone	<0.001	1.78 (1.46–2.16)	<0.001	1.72 (1.40–2.11)
Monobactam	0.397	0.38 (0.04–3.56)	0.564	0.52 (0.06–4.81)
Vancomycin	0.002	1.45 (1.15–1.83)	0.455	1.10 (0.85–1.42)
Other	0.646	0.96 (0.81–1.14)	0.431	0.93 (0.78–0.11)
Length of stay (days)				
1–6	**Ref**	**Ref**	**Ref**	**Ref**
7–13	<0.001	2.69 (2.23–3.25)	<0.001	2.58 (2.13–3.12)
14–20	<0.001	6.70 (5.35–8.38)	<0.001	6.71 (5.34–8.43)
21–27	<0.001	11.78 (9.13–15.21)	<0.001	11.69 (9.00–15.19)
28–34	<0.001	19.02 (14.17–25.53)	<0.001	18.27 (13.47–24.78)
35–41	<0.001	18.88 (13.21–26.97)	<0.001	18.35 (12.65–26.63)
42–48	<0.001	25.40 (17.09–37.76)	<0.001	20.91 (13.59–32.16)
49–55	<0.001	31.45 (19.71–50.18)	<0.001	26.42 (15.97–43.71)
56–62	<0.001	46.93 (27.60–79.80)	<0.001	44.93 (25.83–78.17)
63+	<0.001	57.36 (40.75–80.73)	<0.001	42.08 (29.04–60.97)

a BS, broad spectrum.

b NS, narrow spectrum.

## Discussion

This is one of the largest cohort studies on MRSA acquisition in an acute tertiary-care setting undertaken. Whilst results were consistent with existing literature on significant positive associations between MRSA acquisition and male gender [9], increasing age [10], fluoroquinolone exposure [5,11] and increasing length of stay, the study has also observed factors not previously reported.

The observed association between MRSA acquisition and certain discharging clinical department is an area which may warrant future studies. We hypothesise that the specialties most associated with MRSA acquisition cared for patients that were likely to have compromised skin barriers (dermatology, geriatric medicine and plastic surgery). This is supported by the observation that being discharged from other surgical specialties, where wound surface area is minimised (as a result of wound closure) was either not associated or negatively associated with MRSA acquisition. MRSA acquisition being negatively associated with being discharged from short stay (where length of stay is under 24 h) on multivariate analysis may be due to residual confounding of the length of stay variable.

A possible explanation for the observed negative association between MRSA acquisition and being admitted in the fourth quartile might be the successful introduction of universal MRSA screening and segregation of MRSA-colonised patients from non-colonised patients into different wards for patients admitted through the Emergency Department. Similar large-scale implementations of universal MRSA screening of patients and segregation by MRSA colonisation and infection status have shown similar results [12].

Whilst a history of MRSA colonisation or infection in the year prior to hospitalisation was associated with increased odds of MRSA acquisition (Adj OR 6.39, 95% CI 4.98–8.22), a hospital stay of >20 days had a much larger effect on those odds (21–27 days, Adj OR 11.78, 95% CI 9.13–15.21; 63+ days, Adj OR 57.36, 95% CI 40.75–80.73). The association of fluoroquinolones with MRSA acquisition has been previously reported [13]. However, the results of other studies examining associations with the remaining classes antibiotics have been mixed, making interpretation of the results of the present study challenging [5,11,13]. While further studies may be required to elucidate the mechanism which causes certain antibiotics to be associated with MRSA acquisition, we hypothesise that it may be due to a disruption of commensal flora affecting the ability of MRSA to establish itself on a patient.

This study may be the first to observe a dose–response relationship between length of stay and odds of MRSA acquisition. While acquisition of MRSA infection may be seen as confounding this finding by resulting in a prolongation of length of stay, this dose–response relationship continued to be observed in the sensitivity analysis, which excluded patients who acquired MRSA infection.

Whilst most of the important confounders have been included in the multivariable analysis, residual confounding due to variables not measured and which therefore could not be included in the analysis may be a study limitation. These possible confounders include (a) hand hygiene compliance and MRSA carriage status of healthcare staff, (b) compliance to separation of MRSA-colonised patients from non-colonised patients in MRSA and non-MRSA wards, (c) movement of staff between MRSA and non-MRSA wards, and (d) exposure to indwelling vascular catheters or haemodialysis during admission. Further, while we took into account what specialisation patients were discharged from, we did not examine whether a patient was transferred between specialisations in the time between admission and discharge.

Other limitations of this study include using nares-only swabs for admission screening and pooled swabs for discharge screening, which may have resulted in misclassification, as well as excluding patients with a positive MRSA admission screening result, which did not allow detection of patients who were already colonised with MRSA on admission and subsequently infected with a different pathogenic strain of MRSA.

We were unable to confirm if antibiotics were administered only prior to the detection of MRSA infections among patients who had infections more than 48 h after admission. While this may be a limitation, the sensitivity analysis demonstrates that the dose–response relationship in relation to length of stay in the main analysis persists in the acquisition group.

Nonetheless, this study has several strengths. It is a large cohort study spanning over 2 years and takes into account a larger number of variables at a greater level of granularity than similar studies (especially exposure to antibiotics and surgery, as well as length of stay). The use of data from the structured electronic medical records of patients minimised measurement errors and ensured data accuracy and consistency. None of the data required patient reporting and hence negated the possibility of protocol variation and recall bias.

## Conclusions

This study has identified several important factors associated with MRSA acquisition in a large tertiary-care hospital that has a universal on-admission MRSA screening programme. In spite of the programme requiring the admission of MRSA-colonised and non-colonised patients into separate wards, a strong association between increasing length of hospital stay and MRSA acquisition was observed. This reinforces the importance of facilitating timely discharge planning to minimise length of stay where possible, as well as antimicrobial stewardship and the reinforcement of infection prevention measures including good hand hygiene.
